# Association between ethnicity and hypertension in Northern Colombia in 2015

**DOI:** 10.1186/s40885-022-00203-8

**Published:** 2022-06-15

**Authors:** Drew H. Smith, Jaskaran Grewal, Saba Mehboob, Shiva Mohan, Luisa F. Pombo, Pura Rodriguez, Juan Carlos Gonzalez, Juan Zevallos, Noël C. Barengo

**Affiliations:** 1 Department of Translational Medicine, Herbert Wertheim College of Medicine, Florida International University, Miami, FL USA; 2 American University of Antigua, Osbourn, Antigua and Barbuda; 3 Observatorio de Diabetes de Colombia, Organización para la Excelencia de la Salud, Bogotá, Colombia; 4 Department of Health Policy and Management, Robert Stempel College of Public Health and Social Work, Miami, FL USA; 5 Department of Public Health, Faculty of Medicine, University of Helsinki, Helsinki, Finland; 6 Florida International University Herbert Wertheim College of Medicine, 11200 SW 8th Street, 33199 Miami, FL USA

**Keywords:** Hypertension, Hispanic, Latino, Colombia

## Abstract

**Background:**

Studies in the United States have shown a genetic predisposition to hypertension in individuals of African descent. However, studies on the associations between ethnic groups and hypertension in Latin America are lacking and the limited results have been inconsistent. The objective of this study is to determine whether Afro-Colombian ethnicity increases the risk of hypertension.

**Methods:**

This study is a secondary data analysis of a cross sectional study from five provinces in Northern Colombia. Randomly selected individuals (*N* = 2613; age-range 18–74 years) enrolled in a health care insurance company underwent physical examinations and completed questionnaires regarding ethnicity, lifestyle, and other risk factors. Hypertension in these patients was determined. Unadjusted and adjusted logistic regression analysis were calculated to determine the association between ethnicity and hypertension.

**Results:**

No association between Afro-Colombian ethnicity and hypertension was found (odds ratio [OR], 0.85; 95% confidence interval [CI], 0.66–1.09). As expected, people with a body mass index (BMI) of 30 or higher were at a greater risk of having hypertension (OR, 3.12; 95% CI, 2.35–4.16) compared with those with a normal BMI.

**Conclusions:**

Findings from this study suggest no independent association between Afro-Colombian ethnicity and hypertension. Further research should focus on genotyping or socioeconomic factors such as income level.

## Background

There is convincing evidence from epidemiological studies that elevated blood pressure (BP) is an independent and strong risk factor for cardiovascular diseases, including coronary heart disease and stroke [1, 2]. It has been estimated that worldwide, 7.5 million deaths (12.8% of the global total) and 64.3 million disability-adjusted life years (4.4% of the global total) were due to non-optimal BP [3]. The prevalence of pre-hypertension and hypertension in the Colombian population is estimated to be 62% in the adult population [4].

According to current research findings, a genetic predisposition to hypertension exists particularly in those of African origin in the United States [5]. Nevertheless, studies on the associations between ethnic groups and the risk of hypertension in Latin America has been sparse and have shown inconsistent findings [3, 6]. Evidence from Burroughs et al. [3] found that the prevalence estimates ranged widely from 7 to 49% among reports from Latin America and Caribbean countries. Furthermore, an assessment of national surveys of cardiovascular risk factors in Latin American countries reported that the prevalence of hypertension varied from 12.3% in Colombia to 34% in Argentina [6]. Coincidentally, a longitudinal cohort study of Hispanics in large cities within the United States found significantly different hypertension prevalence throughout five different ethnicity groups [7]. However, the geographic distribution of these studies was not even because they typically only focused on urban areas, which are subject to some selection bias [3, 6].

The purpose of our study was to assess if there are differences between Afro-Colombian ethnicity and non-Afro-Colombians in the prevalence of hypertension in a population sample in the Northern provinces of Colombia in 2015.

## Methods

The participants of this cross-sectional study were the 18–74 years-old population of the Colombian health-care insurance company Mutual SER Entidad Promotora de Salud Subsidiada (Mutual Ser EPSS) living in 30 municipalities in the provinces of Atlántico, Bolívar, Córdoba, Magdalena and Sucre located in northern Colombia. Mutual Ser EPSS is the health-care insurance of the state-subsidized system, thus, providing coverage for those who cannot afford to pay for health services. Mutual Ser EPSS provides healthcare coverage to approximately 1,300,000 people in 5 provinces. The majority of clients registered with Mutual Ser EPSS live in urban regions (77%). All study participants were chosen from the client database of the company through a randomized selection of phone numbers. The sample size was calculated according to an estimated sensitivity of 90% and specificity of 80% to detect new cases of Type 2 diabetes mellitus, considering a confidence level of 95% and an alpha error of 5%. Given an estimated response rate of 70%, the final study sample was 2,550 people [8]. The study sample was calculated for each municipality based on the number of people registered with Mutual Ser EPSS. Thus, the study sample was weighted according to the number of insured Mutual Ser EPSS has in each municipality. The sample was further adjusted proportional to the number of registered clients. Furthermore, to ensure that there were enough participants in each age group, a stratified sample was used with 25% of the participants in age groups of 18–35 years, 36–45 years, 46–54 years and 55–74 years.

The inclusion criteria were defined as individuals between 18 and 74 years old, living in one of five provinces of Colombia (Atlántico, Bolívar, Córdoba, Magdalena and Sucre), who had signed an informed consent. Exclusion criteria included drug treatment for Type 2 diabetes mellitus or a previous diagnosis of diabetes, pregnancy or breast-feeding, history of cancer, regular use of systemic corticosteroids, hemophilia, inability to stand or communicate, and living in areas of difficult access.

All measurements were performed between October 2014 and February 2015 in a clinical healthcare setting. Lifestyle habits and risk factors for Type 2 diabetes mellitus were assessed by an interview using a questionnaire consisting of information regarding ethnicity (which is how categorization as either Afro-Colombian or non-Afro-Colombian was determined), sociodemographic factors, history of Type 2 diabetes mellitus, medical history, tobacco consumption, hypertension, nutritional and physical activity habits. The instruments applied were designed based on the FINDRISC, Stepwise approach to surveillance (STEPS) and International Physical Activity Questionnaire (IPAQ)[9–13].

Height and weight were measured without shoes and with light clothing. Body mass index (BMI) was calculated as weight (kg) divided by height^2^ (m^2^). Waist circumference (to the nearest cm) was measured at the approximate midpoint between the lower margin of the last palpable rib and the top of the iliac crest.

BP was measured in a clinical healthcare setting with mercury sphygmomanometer twice from the right arm of the participant in a seated position after at least 5 min of rest. The mean value of the first and second BP measurements was used in the analysis. Hypertension was defined as a BP greater than or equal to 140/90 mmHg or current treatment for hypertension as per American Heart Association guidelines [14].

### Statistics

Data was analyzed using IBM SPSS ver. 22 (IBM Corp., Armonk, NY, USA) [15]. First, a chi square test was performed to check distribution of the explanatory variables according to exposure, and then outcome. Characteristics between the two ethnic groups were compared using the t-test or the Mann-Whitney for the continuous variables and Chi-squared for the categorical variables. Collinearity diagnostics were performed among all potential confounders to check for multicollinearity in the adjusted model. Univariate and multivariable logistic regression models were used to test the association between ethnicity and hypertension. Odds ratios (OR) and their corresponding 95% confidence intervals (CI) were calculated. A P-value of less than 0.05 was considered as statistically significant.

### Ethical considerations

This study followed the Good Clinical Practice guidelines and the guidelines of the Helsinki Declaration. All data was collected using previously tested questionnaires and methods. Besides blood samples, no invasive methods were used. The study protocol was approved by the Research Ethics Committee of the Central Military Hospital, Bogotá, Colombia (IRB approval number HCM050914). All participants gave written informed consent prior to their participation in the study.

## Results

Table 1 presents the baseline characteristics of the study sample according to ethnicity (Afro-Colombian vs. non-Afro-Colombians) who were living in 30 municipalities of the Northern provinces of Colombia in 2015. There was no statistically significant difference in the distribution of age, sex, educational level, BMI, and prevalence of hypertension and diabetes mellitus between Afro-Colombian and non-Afro-Colombian participants (*P* > 0.05). However, 14% more Afro-Colombians reported having vigorous physical activity compared to non-Afro-Colombians. When comparing smoking status between the two ethnic groups, Afro-Colombians had a significantly higher prevalence to have a history of tobacco use than non-Afro-Colombians in the sample. Afro-Colombians had healthier dietary habits with 46.7% of them consuming at least one fruit or vegetable each day of the week. The corresponding prevalence among non-Afro-Colombians was 37.4% for fruit intake. Afro-Colombians were also less likely to always add salt when preparing food at home, 81.7% vs. 96.9% for non-Afro-Colombians, but were less likely to never add salt to already cooked food, 89.3% compared to 93.8%. Systolic BP was also significantly lower in Afro-Colombians with a median of 115 and interquartile range (IQR) of 100–120 vs. 120 (110–120) for non-Afro-Colombians. While diastolic BP appears quite similar in both groups, with medians of 80 and IQR of 70–80 for each, it is also statistically significant because the non-Afro-Colombian group has a much larger sum of ranks than the Afro-Colombian group and the difference in the sum of ranks is large enough to be statistically significant at the alpha level of 0.05. Clinically, however, there may be no relevant difference.

**Table 1:** Baseline characteristics of the study sample by ethnicity (Afro-Colombian vs. non-Afro-Colombians) in Northern Colombia, 2015

	Ethnicity, n (%)		
**Characteristics**	**Afro-Colombian**	**Non-Afro-Colombian**	**P-value**
Age (yr)			0.087
18–44	261 (40.9)	540 (38.0)	
45–64	257 (40.3)	646 (45.4)	
≥ 65	120 (18.8)	236 (16.6)	
Sex			0.303
Male	253 (39.7)	530 (37.3)	
Female	385 (60.3)	892 (62.7)	
Education			0.296
Uneducated	97 (15.2)	197 (13.3)	
Primary	304 (47.6)	634 (44.6)	
High school	174 (27.3)	425 (29.9)	
Above high school	63 (9.9)	165 (11.6)	
BMI categories (kg/m^2^)			0.086
< 18.5	28 (4.4)	39 (2.7)	
18.5–24.9	248 (39.0)	542 (38.1)	
25–29.9	203 (31.9)	514 (36.1)	
≥ 30	157 (24.7)	327 (23.0)	
Physical activity			< 0.001
High	265 (45.1)	399 (31.7)	
Moderate	167 (28.4)	579 (46.0)	
Low	156 (26.5)	281 (22.3)	
Smoking status			< 0.001
Current	62 (9.7)	87 (6.7)	
Former	150 (23.5)	161 (11.3)	
Never	426 (66.8)	1172 (82.5)	
Addition of salt when preparing food at home			< 0.001
Never	30 (4.7)	12 (0.8)	
Sometimes	87(13.6)	32 (2.3)	
Always	521 (81.7)	1378 (96.9)	
Addition of salt to already cooked food			< 0.001
Never	570 (89.3)	1334 (93.8)	
Sometimes	67 (10.5)	81 (5.7)	
Always	1 (0.2)	7 (0.5)	
Fruits and vegetables consumption (frequency/wk)			< 0.001
At least 1 per day	298 (46.7)	532 (37.4)	
Less than 1 per day	340 (53.3)	890 (62.6)	
Hypertension^a^ (%)	183 (28.7)	440 (30.9)	0.302
Diabetes mellitus (%)	13 (2.0)	32 (2.3)	0.758
Systolic blood pressure (mmHg), median (IQR)	115 (100–120)	120 (110–120)	< 0.001
Diastolic blood pressure (mmHg), median (IQR)	80 (70–80)	80 (70–80)	< 0.001

*BMI* body mass index, *IQR* interquartile range

^a^Blood pressure ≥ 140/90 mmHg, or current treatment for hypertension

**Fig. 1:**
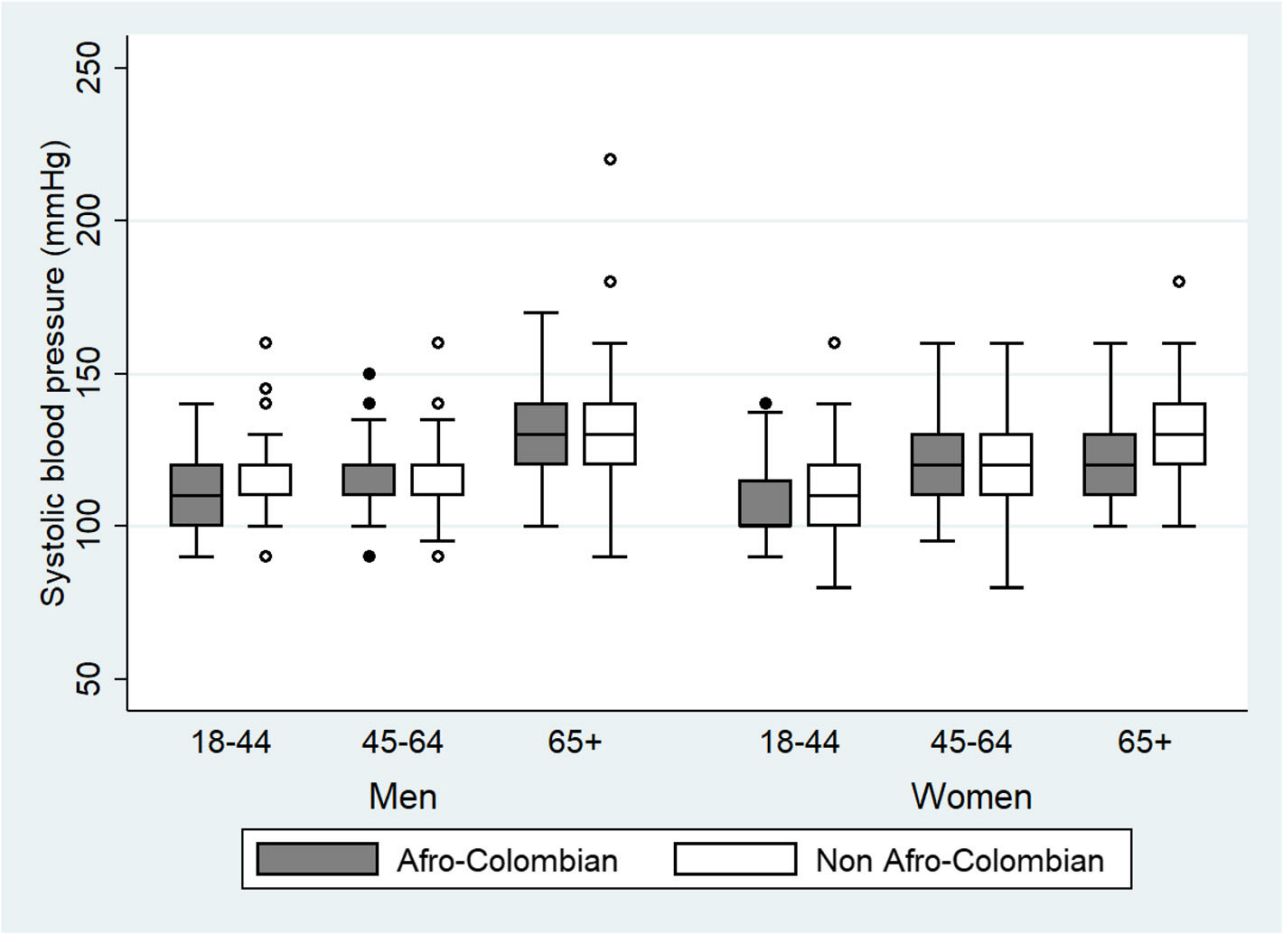
Distribution of systolic blood pressure (mmHg) for Afro-Colombian and Non-Afro-Colombian population of Northern Colombia in 2015 according to sex and age-groups. Side-by-side boxplots show interquartile ranges and medians. Whiskers extend to 1.5 times the interquartile range

**Fig. 2:**
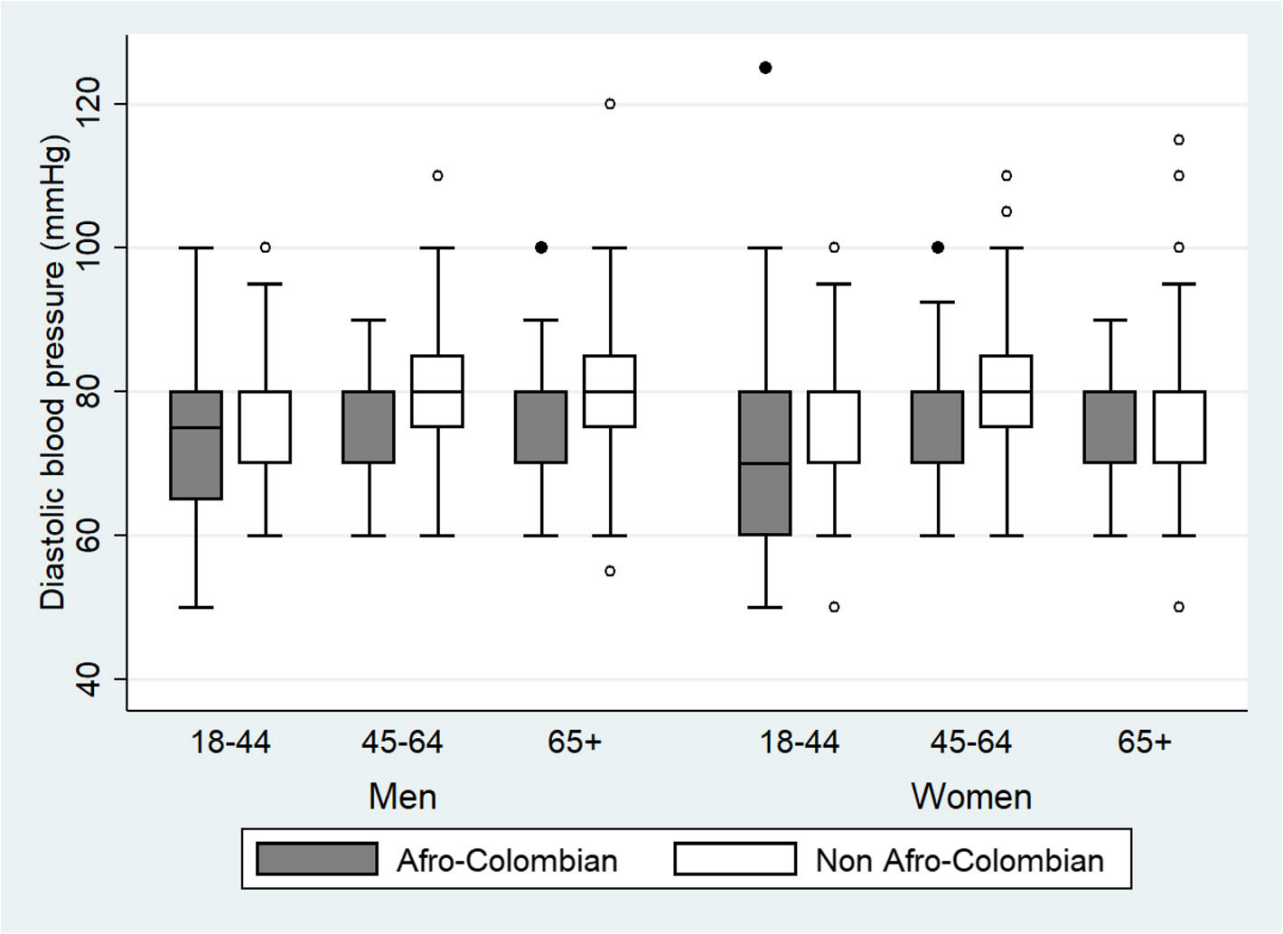
Distribution of diastolic blood pressure (mmHg) for Afro-Colombian and non-Afro-Colombian population of Northern Colombia in 2015 according to sex and age-groups. Side-by-side boxplots show interquartile ranges and medians. Whiskers extend to 1.5 times the interquartile range

Figures 1 and 2 present the distribution of systolic and diastolic BP values for Afro-Colombians and non-Afro-Colombians stratified by sex and age groups. These box and whisker plots show that the median systolic and diastolic BP values were very similar between the Afro- and non-Afro-Colombian population. The median systolic BP values for men were higher in the oldest age-group compared with the younger age-groups. Median diastolic BP values are also similar between Afro- and non-Afro-Colombians.

Table 2 demonstrates the age-adjusted mean values of systolic and diastolic BP distribution in Afro-Colombian and non-Afro-Colombians, including the number of Afro-Colombians and non-Afro-Colombians among categories of ethnicity, women, and men. A total of 638 Afro-Colombians and 1422 non-Afro-Colombians were included. Age-adjusted mean systolic BP was most similar between Afro-Colombian and non-Afro-Colombian men, although it did not vary widely among other characteristics.

**Table 2:** Age-adjusted mean values of blood pressure distribution in Afro-Colombian and Non-Afro-Colombians in Northern Colombia, 2015

		Systolic blood pressure (mmHg)		Diastolic blood pressure (mmHg)	
**Characteristic**	**n**	**Mean**	**95% CI**	**Mean**	**95% CI**
Ethnicity					
Afro-Colombian	638	115.9	114.9–117.0	75.1	74.3–75.9
Non-Afro-Colombian	1422	117.9	117.2–118.6	78.1	77.6–78.6
Female					
Afro-Colombian	385	114.7	113.3–116.1	74.8	73.8–75.8
Non-Afro-Colombian	892	117.4	116.5–118.2	77.7	77.1–78.4
Male					
Afro-Colombian	253	117.7	116.1–119.2	75.5	74.4–76.7
Non-Afro-Colombian	530	118.9	117.8–120.0	78.7	77.9–79.5

*CI* confidence interval

Table 3 shows the unadjusted and adjusted associations between various covariates and the risk of hypertension in study participants living in 30 municipalities of Northern Colombia. There was no statistically significant association in the unadjusted model between race/ethnicity and hypertension (OR, 0.90; 95% CI, 0.73–1.10). After controlling for several potential confounders, the OR remain very similar (OR, 0.85; 95% CI, 0.66–1.09).

**Table 3:** Unadjusted and adjusted associations between various covariates and the risk of hypertension in Northern Colombia in 2015

Characteristics	Unadjusted	Adjusted
	**OR (95% CI)**	**OR (95% CI)**
Ethnicity		
Non-Afro-Colombian	Reference	Reference
Afro-Colombian	0.90 (0.73–1.10)	0.85 (0.66–1.09)
Age (yr)		
18–44	0.19 (0.15–0.26)	0.22 (0.17–0.30)
45–64	Reference	Reference
≥ 65	3.03 (2.35–3.90)	3.59 (2.69–4.77)
Sex		
Female	Reference	Reference
Male	1.09 (0.90–1.33)	1.17 (0.92–1.49)
Education		
Uneducated	2.58 (1.74–3.84)	0.88 (0.55–1.41)
Primary	2.17 (1.53–3.07)	0.96 (0.64–1.43)
High school	0.95 (0.65–1.39)	0.78 (0.51–1.20)
Above high school	Reference	Reference
BMI^a^ categories (kg/m^2^)		
< 18.5	0.35 (0.15–0.83)	-
18.5–24.9	Reference	Reference
25–29.9	1.76 (1.40–2.22)	1.74 (1.34–2.25)
≥ 30	2.71 (2.12–3.47)	3.12 (2.35–4.16)
Physical activity		
Low	1.38 (1.03–1.84)	1.15 (0.81–1.62)
Moderate	1.52 (1.10–2.09)	1.22 (0.84–1.77)
High	Reference	Reference
Smoking status		
Never	Reference	Reference
Former	1.97 (1.54–2.53)	1.03 (0.76–1.40)
Current	0.78 (0.53–1.15)	0.55 (0.35–0.87)
Addition of salt when preparing food at home		
Never	1.93 (1.04–3.57)	1.71 (0.83–3.54)
Sometimes	0.97 (0.65–1.46)	0.93 (0.58–1.50)
Always	Reference	Reference
Addition of salt to already cooked food		
Never	Reference	Reference
Sometimes	0.61 (0.41–0.92)	0.69 (0.44–1.09)
Always^b^	-	-
Fruits and vegetables consumption (frequency/wk)		
At least 1 per day	Reference	Reference
Less than 1 per day	0.84 (0.70–1.02)	0.85 (0.68–1.06)
Diabetes mellitus		
No	Reference	Reference
Yes	1.41 (0.77–2.60)	1.17 (0.59–2.33)

*OR* odds ratio, *CI* confidence interval, *BMI* body mass index

^a^Underweight category was not included, only 6 participants were underweight and reported hypertension. ^b^Not included because only 8 participants selected the option: “always add salt to foods after the food was cooked or when they sit at the table”

Participants older than 64 years (OR, 3.59; 95% CI, 2.69–4.77) had a statistically significant higher odds of hypertension compared with 45–64 years-old participants.

Moreover, a statistically significant association was found between patients classified as obese based on their BMI (BMI ≥ 30 kg/m^2^) and the odds of hypertension. Individuals with obesity had a nearly three-fold increased odds to develop hypertension compared with those with a normal BMI between 18.5 and 24.9 kg/m^2^ (OR, 3.12; 95% CI, 2.35–4.16). Adjusted associations between sex, education, physical activity, smoking status, addition of salt when preparing food at home, addition of salt to already cooked food, fruit and vegetable consumption, diabetes mellitus and hypertension were not statistically significant.

## Discussion

Our study did not find an increased odds of hypertension in Afro-Colombians compared with non-Afro-Colombians.

While predominately an epidemiological study, explanations may lie within the growing field of genetic epidemiology, which scrutinizes the role of inherited factors to comprehend disease etiology [16]. Several mechanisms have been proposed to genetically predispose African Americans to hypertension. Africans have an increased sensitivity to salt, in addition to suppression of plasma renin, which leads to subsequent higher kidney natrium retention. This heritable feature may be explained by the dry and warm climates that dominate in the African regions, many of which are scarce in salt [17]. Plasma renin and systolic BP have demonstrated an inverse relationship in normotensives. Blacks are two times more likely than whites to have a low-renin form of hypertension, with high levels of aldosterone that are not proportionate with renin levels [18]. However, the mechanisms of salt sensitivity are polygenetic and multifactorial, and probably fluctuate between and even within ethnic and geographic populations [19].

Conflicting results have been published in regards to the associations between ethnicity/race and hypertension in Latin America [5–7, 20, 21]. Cooper et al. [5] found that South-Africans had significantly higher systolic BP when compared to participants in the US, Jamaica, and Ghana. They were also able to confirm prior trends showing increased prevalence of hypertension in populations of African-origin amongst lower socioeconomic, multi-racial societies [5].

Moreover, studies conducted in the United States among Hispanics found non-Hispanic blacks to have the highest rates of hypertension among many subgroups in New York City [22]. Giambrone et al. [20] found very similar results when comparing non-Hispanic blacks to Hispanic and Asian subgroups. They found non-Hispanics blacks to have an approximately 5% increased prevalence of hypertension. In fact, in 2010, when Pabon-Nau et al. [21] analyzed the results of the National Health Interview Survey, country of origin played a significant factor in prevalence of hypertension. Furthermore, higher rates of hypertension were noted amongst Hispanic immigrants from countries with higher black populations [21].

The heterogeneity of the Colombian population could be the main explanation for the lack of association between ethnicity/race and hypertension. The Colombian population has a significant admixture from three ancestries, European, Indigenous Native American and African, and the percentage difference diverges substantially in the various regions of the country [23]. Similarly, studies completed in Cuba during 2005 and 2013 found equal rates of hypertension among blacks and white [19, 20]. The heterogeneity of the Cuban population may have also contributed to the results. Nevertheless, our sample’s heterogeneity may be subject to a degree of classification bias as it relies on self-reported binary labeling as Afro-Colombian or non-Afro-Colombian and further investigation using genotyping could be beneficial.

Our findings are in agreement with the prevalence of hypertension reported in several populations living in countries in Latin American despite their heterogeneous population [5, 24, 25]. After adjustment, the factors of age, BMI, smoking status, diet (fruit/vegetable consumption), and physical activity showed the expected logical relationship with hypertension onset [26–29].

The lack of association between ethnicity and hypertension onset in our sample leads us to focus on other factors such as socioeconomic status and income inequality. These factors have been shown to play a contributory factor in hypertension [4]. Additional follow-up examining income level may provide further insight into the role on hypertension of Latin American nations. Naturally, other factors such as the progression of vascular stiffness due to age, lifestyle, and diet are central to the development of hypertension and may supersede any genetic liability.

Finally, one explanation for not finding a statistically significant association is lack of power (type 2 error). In order to see assess whether our study had sufficient power to detect a statistically significant association between ethnicity and hypertension we applied several methods. The estimated strength of the association was not large. However, the lower bound of the CI extend to values that could be clinical important although the 95% CI includes 1 (no statistically significant association). Thus, based on this estimation the study cannot clearly rule out a potential important effect. We also conducted post-hoc power analysis. Given a two-sided CI of 95%, the post-hoc power analysis revealed a power of 16% to detect a difference of 2.2% in hypertension prevalence between the two groups. The post-hoc power analyses are typically calculated on negative results (P ≥ 0.05), thus producing a low power result. There are many critical discussions currently ongoing about the use, benefit, and information provided by the post-hoc power analysis and the assumptions involved in the post-hoc power analysis. In summary, type 2 error may be the most likely reason not to find a statistically significant association in our study.

The study has some limitations. The survey relied on self-reported answers, which are subject to recall and social desirability bias and may affect results. As with any cross-sectional study, we cannot assess the temporal sequence of the association between any of the risk factors studied and hypertension. Moreover, research carried out by Sorlie et al. [7] found that almost 50% of adult Hispanic males in the Study of Latinos study in the United States were not aware of their hypertension. This percentage may be even higher in a developing country such as Colombia. Thus, relying on partially self-reported data may have underestimated the proportion of hypertensive patients in Colombia. Other limitations in our study include the inability to measure salt intake using the gold standard of 24-hour urine samples and the exclusion of patients with known diabetes regardless of hypertension status. This may have resulted in fewer hypertensives participating in our study and may be underestimating our association. Even though the study was originally designed to develop a risk score for type 2 diabetes, sufficient information was collected from the study participants to test several research questions related with hypertension. Unfortunately, information on additional laboratory tests such as 24-hour urine samples to measure sodium concentration was not available. Finally, this is a study of a specific population located in a specific urban geographic region. The Afro-Colombian population has been noted to have strong admixture with native Indians; this is reflected in studies of haplotypes of the Colombian population [30]. As a result, extrapolations of our study may not be applicable to other regions. However, it does highlight the possible need for genotyping when evaluating associations between ethnicity and hypertension and the impact of population heterogeneity, which could impact hypertension studies not just in South America, but also other areas of the world.

## Conclusions

In conclusion, our findings suggest that there was no association found between Afro-Colombians and hypertension in Northern Colombia. A number of factors may be contributing to this lack of association, including heterogeneity and socioeconomics. Further research should focus on additional contributing factors to hypertension, including social environmental factors such as income level. Repeating the study after genotyping each participant could also be quite revealing. Although no association between ethnicity and hypertension was found, an average of 4 of 10 non-diabetic participants were affected by hypertension in this study. We continue to advocate for regular hypertension screenings regardless of ethnicity or any other factor.

## Data Availability

The datasets used and/or analyzed during the current study are available from the corresponding author on reasonable request.

## Abbreviations

BMI: Body mass index
BP: Blood pressure
CI: Confidence interval
IQR: Interquartile range
OR: Odds ratios
